# Pioneering insights of extrachromosomal DNA (ecDNA) generation, action and its implications for cancer therapy

**DOI:** 10.7150/ijbs.73479

**Published:** 2022-06-13

**Authors:** Zesheng Li, Bo Wang, Hao Liang, Lei Han

**Affiliations:** Tianjin Neurological Institute, Key Laboratory of Post-Neuroinjury Neuro-repair and Regeneration in Central Nervous System, Ministry of Education and Tianjin City, Tianjin Medical University General Hospital, Tianjin, 300052, P.R. China.

**Keywords:** extrachromosomal DNA (ecDNA), cancer, oncogene amplification, function of ecDNA, tumour evolution, intratumoural heterogeneity

## Abstract

Extrachromosomal DNA (ecDNA) is a cancer-specific circular DNA molecule that is derived from chromosomes. In contrast with linear chromosomes, ecDNA exhibits a unique structure that can be representative of high chromosome accessibility, contributing to hyperactivated proto-oncogenes and malignant behaviours. Meanwhile, nonchromosomal inheritance and recurrent mutations of ecDNA fuel tumour heterogeneity and evolution. Recent studies have demonstrated that ecDNA drives tumorigenesis and progression and is related to poor clinical outcomes and drug resistance across widespread cancers. Although ecDNA was first observed in 1965, with technological advancements, its critical functions in tumorigenesis are currently coming forth. In this review, we summarize the current understanding of the origin, biogenesis process, discovery history, molecular mechanisms, and physiological functions of ecDNAs in cancer. Additionally, we highlight the effective research methods to study ecDNA and offer novel insights for ecDNA-directed therapies.

## Introduction

Eukaryotic genomic DNA is packaged into linear chromosomes. For various reasons, some genomic DNA can separate from the chromosomes and form particles of different sizes. Biophysical methods and DNA sequencing confirmed that these particles were circular, and thus, they were named extrachromosomal circular DNA (eccDNA). They can encode regulatory elements (promoter elements, enhancer elements, etc.) and genes. To date, eccDNA particles have been found in a variety of eukaryotes, including yeast 1, drosophila 2, c. elegans 3 and humans 3-6. EccDNAs exert effects on several aspects, including cell phenotype, heterogeneity and response to environmental stress. Therefore, revealing the characteristics of eccDNAs in diseased tissues might advance the diagnosis of diseases and improve the current therapies. Additionally, eccDNAs can be released as extracellular free DNAs from tissue cells into the biological fluid. So, eccDNAs might serve as novel biomarkers to shed new light on the improvement of early detection and the monitoring of responses to drug treatments. According to the size of the eccDNA, these particles can be divided into four main types, namely, small polydispersed circular DNA (spcDNA, several hundred bp) 1, microDNA (100 to 400 bp) 7-9, telomeric DNA (t-circles/c-circles, 738 bp to multiples of 738 bp) 10, and extrachromosomal DNA (ecDNA, 1 to 3 Mb) 9. Recently, it has been reported that oncogene-carrying extrachromosomal DNA (ecDNA) exhibits more correlation with tumour pathogenesis than other types of eccDNAs 11. In this perspective, we focus on the role of ecDNA, highlighting its importance to tumour pathogenesis and cancer evolution.

Extrachromosomal DNA is also known as double minutes (DMs) 12. DMs are paired spherical chromatin bodies with several mega-base pairs of size which are representative of gene amplification. New research has revealed that ecDNA is closely related to the amplification of oncogenes and the increase in intratumor heterogeneity. The unique circular structure might qualify the ecDNA with more accessible chromatin that contributes to oncogene amplification 13-15. On the other hand, ecDNA lacks centromeres, resulting in the different copy number of ecDNA in daughter cells. The inheritance patterns can enhance tumour heterogeneity 9, 16, 17. Interestingly, subsequent studies have also found that ecDNA is crucial for chemotherapy resistance in tumours 18. Studies on the biological functions of ecDNA are still in their infancy and have attracted increasing attention. In this article, we mainly focus on the structural characteristics, functional mechanisms, detection methods and clinical application of ecDNA in tumours. Future directions for the field, including druggable ecDNA targets, considerations for bringing ecDNA biomarkers to practice, and cancer-specific ecDNA will be addressed.

## Exploration history of ecDNA

**Figure 1:**
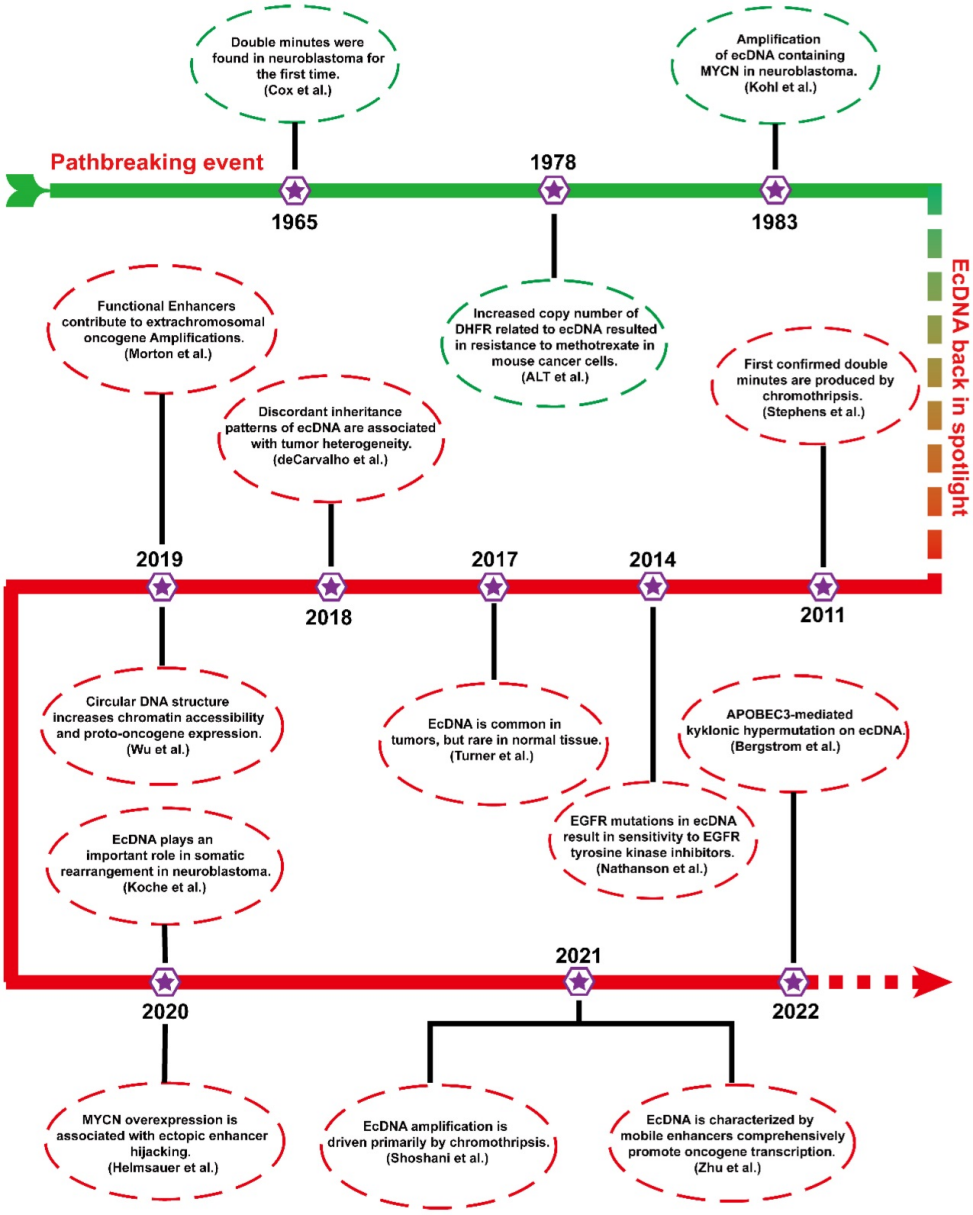
** Timeline of landmark ecDNA explorations.** This timeline emphasizes several vital findings of ecDNA, which will contribute to a better understanding of ecDNA in tumours.

Before engaging in ecDNA research, it is necessary to understand some of the pioneering events of ecDNA. **Figure 1** shows the timetable for several milestones of ecDNA.

In 1965, ecDNAs were found in metaphase neuroblastoma as small chromatin bodies 12. As they often appear in pairs, they are also called “double minutes” (DMs).

From the late 1970s to the early 1980s, scientists conducted a landmark molecular-level study. ALT et al. first found that an unstable copy number increase in the dihydrofolate reductase gene (DHFR) resulted in resistance to methotrexate in mouse lymphoma cells, which was associated with ecDNA^18^. To date, ecDNA containing different oncogenes (MYCN and MYC) has been identified in different tumours 19, 20. The biogenesis and progression of tumours are closely related to the amplification of ecDNA harbouring oncogenes.

More than 20 years later, ecDNA has returned to the public view, and studies are gradually emerging. In 2011, the formation of ecDNA during chromothripsis was thoroughly elucidated via next-generation sequencing technology 21. Chromothripsis refers to breakage events in which a single chromosome is shattered into tens to hundreds of fragments. Then, these chromosomal fragments are religated randomly by DNA double-strand break repair. Chromothripsis can remodel the cancer genome by inserting or deleting DNA fragments that often trigger copy number alterations, oncogenic gene fusion, and tumor suppressor gene inactivation 22, 23. In 2014, a study confirmed an unexpected phenomenon that EGFRvIII mutations were mainly localized in ecDNA 24. In 2017, Turner's team reported that ecDNA was ubiquitous in tumours and present in almost half of all human tumours but rare in normal cells 14. Soon after, further studies found that uneven inheritance patterns of ecDNA can affect the carcinogenic potential of cells with ecDNAs 25. Uneven segregation of ecDNA during mitosis leads to different ecDNA copy numbers in daughter cells, which results in genomic heterogeneity of tumour cells and favors the dynamic evolution of cells with higher copy numbers. In 2019, it has been found that some functional enhancers from adjacent regions of ecDNA coamplified with oncogenes 26. In the same year, Wu et al. found that the expression level of oncogenes in ecDNA is among the highest in the tumour transcriptome and that the circular structure of ecDNA enhanced its chromatin accessibility and oncogene expression 11.

In the last two years, increasing breakthroughs have been achieved in ecDNA. Recent studies have shown that ecDNA is capable of driving somatic rearrangement in neuroblastoma, and thus is a key genomic feature of cancer 27. Helmsauer et al. identified the structure of ecDNA containing MYCN using short-read and nanopore sequencing and analysed its chromatin landscape. They revealed that MYCN overexpression is associated with enhancer hijacking 28. Shoshani's team studied chemotherapy-resistant clonal cell isolates using whole genome sequencing and found that ecDNA amplification is driven primarily by chromothripsis 23. Recent study revealed that ecDNAs can serve as mobile super-enhancers (SEs), which fuels genome-wide transcriptional activity, including that of oncogenes. This finding confirms that ecDNA can promote tumorigenesis by interacting in trans with chromosomal genes 29. Recent studies have reported a novel clustered somatic mutations event termed kyklonic hypermutation, which was frequently observed on ecDNA. The kyklonic hypermutation might account for the evolution of tumour subclones 30.

## The generation mechanism of ecDNA

The biogenesis of ecDNA is not exactly the same as that of eccDNA. There are many pathways of ecDNA biogenesis, and each pathway is accompanied by DNA damage and loss of tumour suppressor genes.

### Chromothripsis

**Figure 2:**
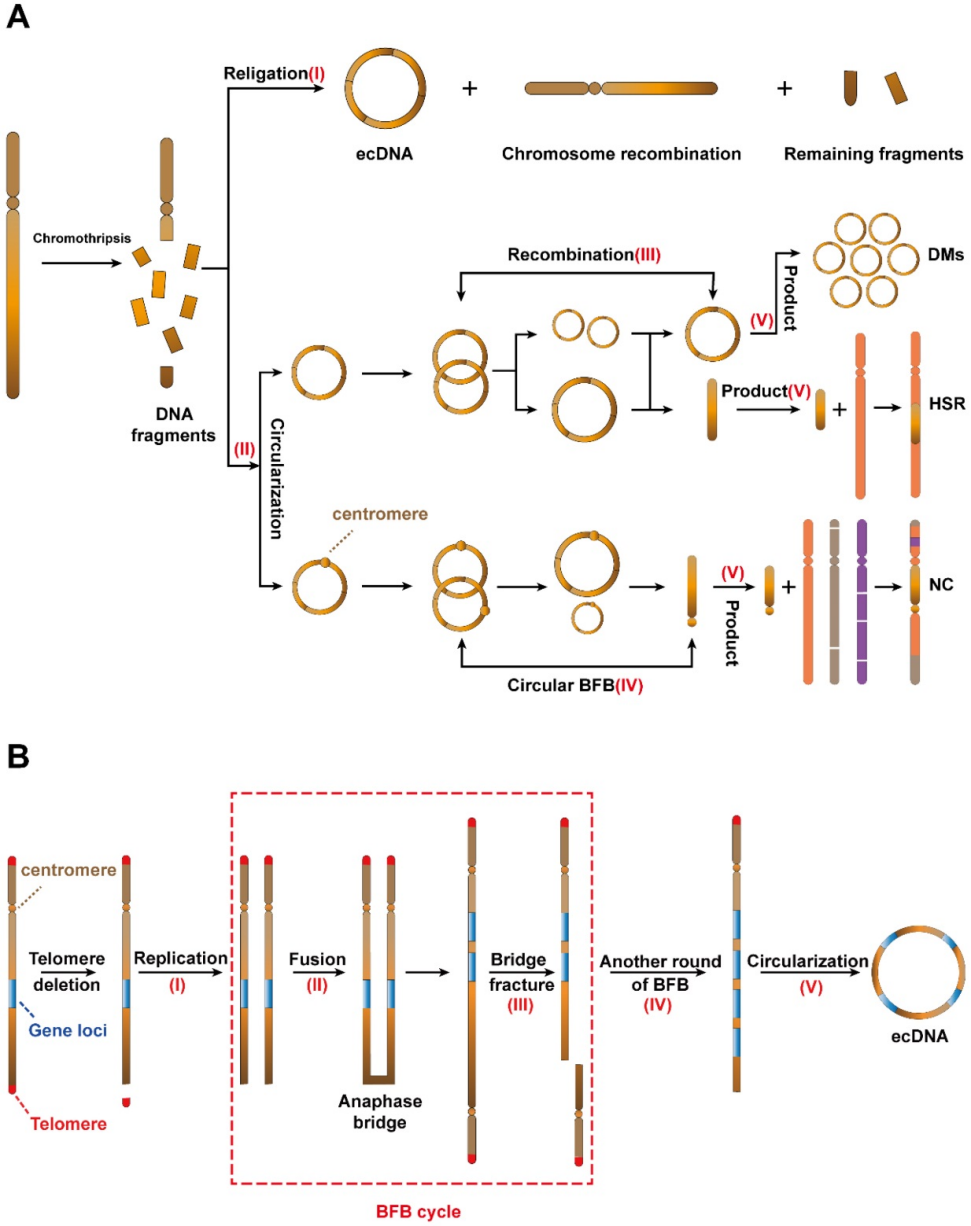
**Potential mechanisms of ecDNA biogenesis. (A)** Chromothripsis: (I) DNA fragments from chromothripsis religate to form ecDNA. (II) The DNA fragments are initiated by chromothripsis, and its circularization involves the evolution of seismic amplification. Seismic amplification is initiated by chromothripsis. DNA fragments from chromothripsis are then circularized. The circular DNA fragment without centromeres is amplified, undergoes circular recombination and forms a large circular fragment or a linear fragment. While the large circular fragment evolves to double minutes (DMs), the linear fragment evolves to homogeneous staining regions (HSRs). On the other hand, the circular DNA fragment with the centromere undergoes circular BFB cycles and forms a linear fragment with the centromere. Eventually, the linear fragment with centromeres forms neochromosomes (NC). **(B)** The breakage-fusion-bridge (BFB) cycles model: A dicentric anaphase bridge, formed due to the deletion of telomeres, is broken into fragments under stress. The fragments harbouring gene loci will either continue to replicate to form a new dicentric anaphase bridge or loop out to form ecDNA.

As the most common mechanism for ecDNA biogenesis, chromothripsis occurs when a chromosome suffers catastrophic DNA damage and breaks into several DNA fragments of varying sizes. These DNA fragments then rearrange. The above process acts as a strong driver of tumorigenesis^31^. However, in some cases, these DNA fragments can be religated and circularized to form ecDNA (I) **(Figure 2A, top)** 21, 31-33.

In 2021, Rosswog et al. proposed a new amplification pattern that is completely different from other amplification patterns, namely, “seismic amplification”. More interestingly, they found that this amplification pattern is a dynamic evolution process and is closely related to chromothripsis **(Figure 2A, bottom)**. The process begins with the chromothripsis of one or more chromosomes. Then, these fragments reassemble into circular DNA structures (II). Third, these circular DNAs without centromeres undergo sequence recombination to form large circular DNA fragments or linear fragments (Ⅲ). On the other hand, these circular DNAs with centromeres form linear fragments with centromeres through the circular breakage-fusion-bridge (BFB) cycles (IV), which is a BFB cycles variant that acts on circular ecDNA (the BFB cycle is a type of chromosomal behavior in which a broken chromatid fuses to its sister, thus forming a “bridge”. When the centromeres separate at mitosis, the chromosome breaks again, thereby restarting the cycle). Eventually, these fragments participate in the next stage of evolution and form three products (Ⅴ), namely, double minutes (DMs), which retain the circular structure; homogeneous staining regions (HSRs), which are generated by the integration of the circular sequence into the chromosome and neochromosomes (NC), which refers to the aberrant chromosome 34.

Recurrently chromothripsis is the driving factor leading to drug resistance of tumour, but DNA repair pathway is necessary for recombination of chromothripsis. Therefore, we suspect that the development of combination therapy targeting these processes may significantly reduce tumour resistance and improve patient outcomes.

### Breakage-fusion-bridge (BFB) cycles

The breakage-fusion-bridge (BFB) cycles, a mechanism for ecDNA biogenesis 35, begins with the deletion of telomeres on a single chromosome and the replication of chromosomes which are missing telomeres (I). The two DNA sequences missing telomeres then fuse to form a new chromosomal structure with two centromeres and an anaphase bridge (II) 36. The synthetic new chromosome holds two centromeres, and bridge fracture can be divided into two daughter cells (III). Gene loci in one bridge fracture will duplicate in daughter cells. The above process can iterate multiple times (IV) and the products will eventually be recombined to form ecDNA (V) **(Figure 2B)** 35.

### Slight damage to DNA and religation

Studies of ecDNAs and their chromosomal origin have revealed that ecDNAs might originate from slight DNA damage. In the 1980s, because of the lack of available detection methods, it was difficult to achieve comprehensive genomic profiling. Therefore, Wahl et al. utilized Southern blotting to explore the dynamics of episomes (an extrachromosomal piece of genetic material) in an analogue system. They demonstrated that the biogenesis of ecDNA was related to the deletion of chromosome fragments 37. Recently, a study reported that ecDNA containing MYC (ecMYC) was amplified in leukaemia samples, which was uncovered by fluorescence in situ hybridization (FISH). Surprisingly, deletion of the MYC locus was also detected on chromosome 8, which further supported that the deletion of chromosomal MYC is responsible for the ecMYC product 38.

**Figure 3:**
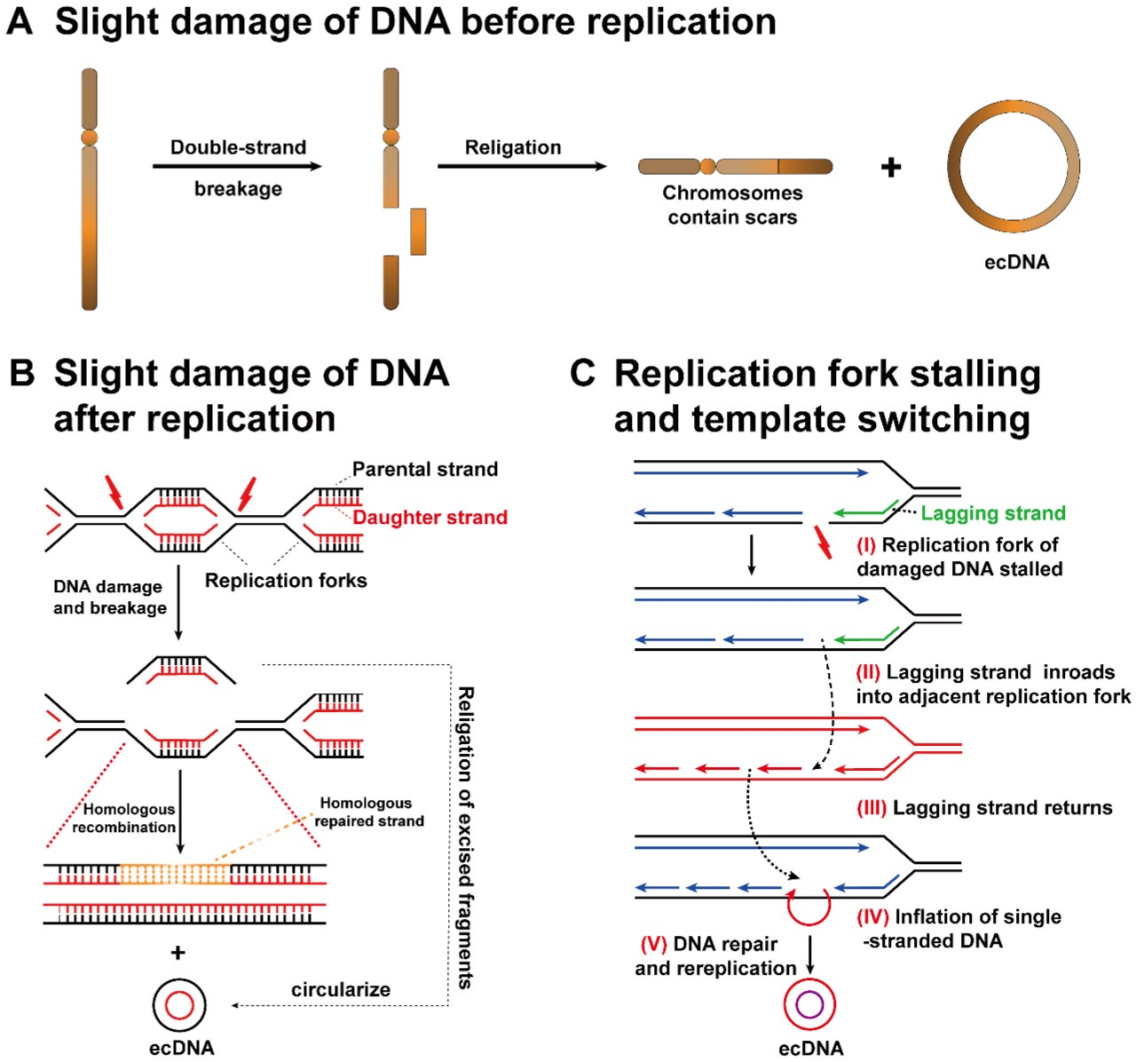
**Potential mechanisms of ecDNA biogenesis. (A)** Slight DNA damage before replication: one arm of a chromosome breaks, and then the DNA fragment that separates from the original chromosome forms ecDNA. The original chromosome leaves a scar. **(B)** Slight DNA damage after replication: if slight DNA damage occurs between two replication forks, the detached double-stranded DNA is circularized to form ecDNA, while chromosomes are repaired by a homologous recombination mechanism. **(C)** Replication fork stalling and template switching: DNA damage occurs in the template strand, and then the lagging strand stalls. The lagging strand detaches from the original template strand, inbreaks adjacent replication forks, and then continues to participate in new DNA synthesis. Strand detachment and inbreaking could occur in many rounds until the strand returns to the original template, resulting in the inflation of single-stranded DNA in either strand. Ultimately, the inflated single-stranded DNA replicates into a double-stranded circular DNA structure.

More importantly, it is also possible that the origin chromosome has no missing gene locus matching ecDNA fragments. The above findings suggest that ecDNA biogenesis may occur in two aspects: (1) EcDNA consists of fragments that have fallen off from identical or dissimilar chromosomes. The original chromosome will exhibit a scar **(Figure 3A)**. (2) The sequence of double-stranded DNA is cut off between two replication forks and then forms ecDNA. The scars left on the replication fork can be healed by homologous replication **(Figure 3B)**^39^, ^40^.

### Replication fork stalling and template switching

Through the analysis of the ecDNA breakpoint sequence, a possible mechanism related to ecDNA biogenesis, replication fork stalling and template switching was clarified 41. The DNA replication fork stagnates at the breakpoint (I). The lagging strand separates from the present template strand, makes inroads into the active adjacent replication fork, and then participates in the synthesis of new DNA (II). Lagging strand intrusion and recombination may occur repeatedly until the strand finally returns to its template strand (III). There is no doubt that the original template is not completely complementary to the newly formed DNA strand during template switching, resulting in the inflation of single-stranded DNA in either strand (IV). Ultimately, the inflated single-stranded DNA replicates to form a double-stranded circular DNA structure (V). Single-stranded DNA cannot be ruled out as a source of ecDNA, although the biological mechanism of ecDNA has not been firmly established **(Figure 3C)** 42.

### Instability of chromosome DNA and ecDNA

New research has begun to focus on the genetic background of ecDNA. It is well known that the loss of tumour suppressor genes is a manifestation of genomic instability. Smolen et al. found that deletion of tumour suppressor genes, including Trp53 and Brca1, plays an integral role in the amplification of the ecDNA-containing oncogene MET (ecMET) in mouse breast cancer cells 43.

Although ecDNA has been formed, it keeps evolving. Previous studies have suggested that the larger ecDNA is formed by episomes expanding in cells 37. Later studies found that episome expansion was not indispensable for ecDNA formation 44. Therefore, there may be other mechanisms involved in ecDNA formation. In this way, the structure and sequence of ecDNA could change over time. For example, new ecDNA found in recurrent neuroblastoma was formed by integrating new fragments into the ecDNA found in the primary tumour 45.

## The characteristics of ecDNA

### Circular characteristics of ecDNA

To date, research on the structure of ecDNA remains the focus of much attention. Previous ultrastructural analyses have elucidated some fundamental characteristics of ecDNA by transmission electron microscopy (TEM). First, ecDNA consists of chromatin harbouring nucleosomes, which are then woven into a typical chromosomal structure. Second, studies confirmed that there are no free sequences of ecDNA, and that its structure seems to be circular 46-48. In the late 20^th^ and early 21^st^ century, the ultrastructure of ecDNA was further explored by scanning electron microscopy (SEM) and atomic force microscopy (AFM). Nevertheless, owing to the limited resolution of SEM and AFM, the observed images were not consistent with the true structure of circular ecDNA 49-51.

In 2019, a study confirmed that ecDNA exhibits a circular structure by DNA sequencing, ultrastructural imaging and long-range optical mapping. First, WGS with amplicon architecture analysis expounded the circular structure developed by some DNA fragments with breakpoint religations between them. Second, long-range optical mapping clarified a consecutive contig that steps over all breakpoints of the ecDNA and confirmed the circular structure of ecDNA. Third, these ultrastructure images, including SEM, TEM and three-dimensional structured illumination microscopy, indicate that the ecDNA is unequivocally circular 11.

### ecDNA suffered a mass of mutations

Cancer genomes feature a mass of somatic mutations that qualify cancer cells with survival advantages. Earlier studies have reported some clustered somatic mutations, such as clustered single-base substitutions, diffuse hypermutation (termed omikli), and longer strand-coordinated events (termed kataegis). Recently, Bergstrom et al. observed multiple kataegis hypermutation on ecDNA (termed kyklonic hypermutation) 30.

**Figure 4:**
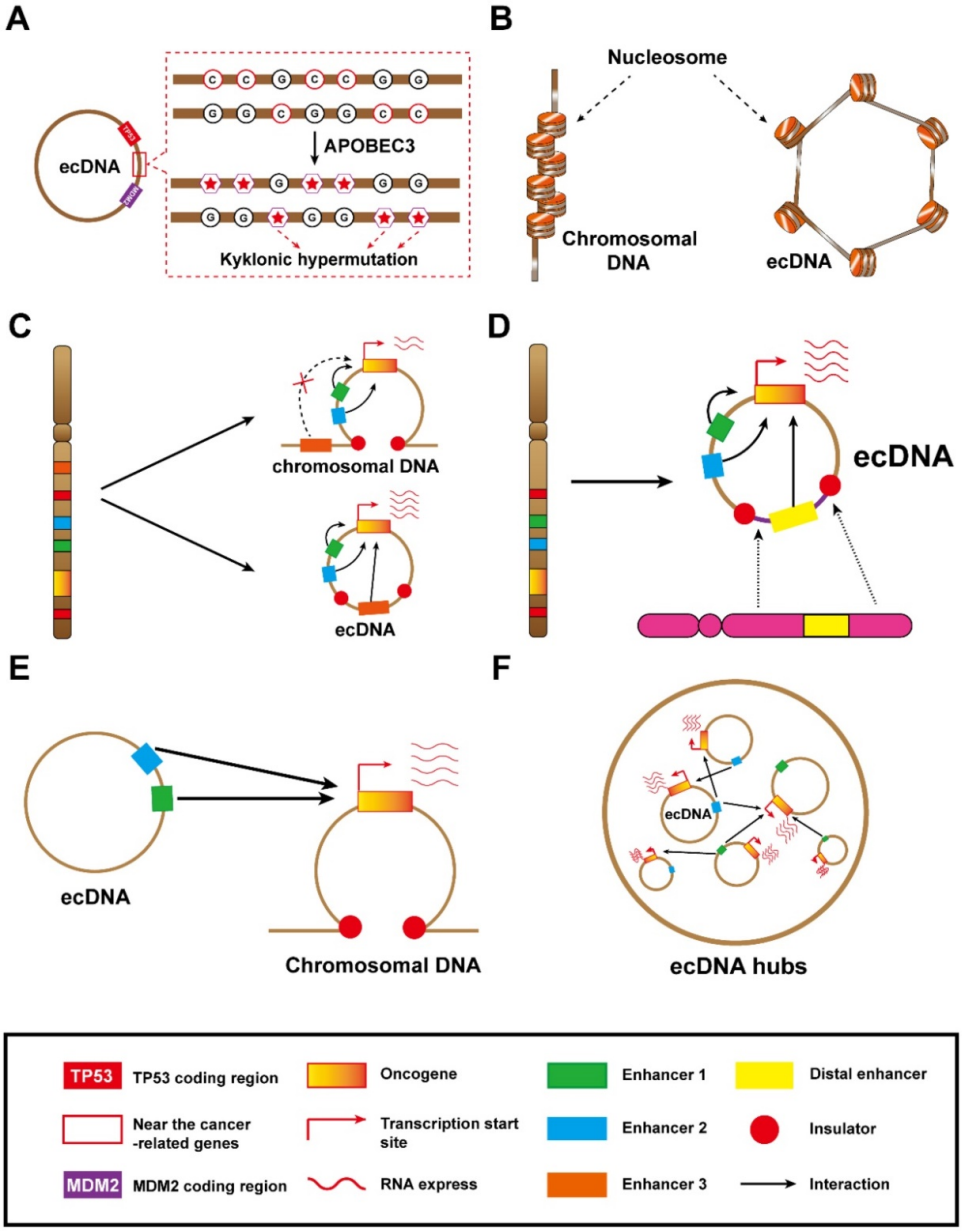
**The unique structure and molecular mechanisms of ecDNA. (A)** This ecDNA suffered a mass of mutations (kyklonic hypermutation) near the cancer-associated genes: ecDNA was treated as an infectious agent and attacked by APOBEC3 enzymes. **B)** ecDNA is circular: the nucleosomal organization of ecDNA is less compact than that of chromosomal DNA, coupled with a highly accessible epigenetic landscape. These features lead to increased transcriptional activity and subsequent increased expression of amplified oncogenes in ecDNA. **(C)** Local enhancer hijacking: an enhancer of an adjacent topologically associating domain is combined with the oncogene into a circular domain. **(D)** Distal enhancer hijacking: a distal enhancer sequence is integrated with the oncogene into a circular domain. **(E)** ecDNA-chromosomal DNA interaction: ecDNA can act in trans with chromosomal DNA. **(F)** ecDNA hubs and ecDNA-ecDNA interaction: ecDNA spatially clustered with other ecDNAs, forming ecDNA hubs that prompt intermolecular regulation among ecDNAs.

ecDNA containing cancer-related genes often suffers kyklonic hypermutation, which fuels the evolution of ecDNA. A higher frequency of kyklonic hypermutation in ecDNAs loaded with cancer-related genes has been observed 30. Moreover, recurrent kyklonic hypermutation was increased within or near cancer-related genes, including ARNT, TP53 and MDM2 **(Figure 4A)**. Importantly, recurrent kyklonic hypermutation has been observed across widespread cancers, including glioblastomas, lung cancers and other malignant cancers 30, 52.

Additionally, they also revealed that the kyklonic hypermutation within ecDNAs was dominated by APOBEC3 30. This APOBEC3-associated kyklonic hypermutation contributed 97.8% of all kyklonic hypermutation, and more than 30% of ecDNAs had one or more kyklonic hypermutation. APOBEC3 is a critical host protein that prevents the replication of retroviruses by mutating the viral genomes. In this way, the viral-like circular structure of ecDNA is prone to be treated as a virus and incurs attacks from APOBEC3 enzymes, which induce kyklonic hypermutation on ecDNA 30. APOBEC3-associated kyklonic hypermutation on ecDNA may be related to tumour evolution, evasion of therapies and clinical outcomes. Thus, further analysis of large-scale clinical data for multiple cancers is required to deeply explore the clinical significance of kyklonic hypermutation. In addition, APOBEC3-mediated kyklonic hypermutation frequently occurs in ecDNA, and this process undergoes extensive recombination of DNA fragments 30. Therefore, we concluded that APOBEC3-mediated kyklonic hypermutation may also be a cause of chromothripsis. And this new tumorigenesis model also lays the foundation for new therapeutic models. Developing drugs that limit APOBEC3 activity may be a boon for cancer patients.

## Molecular mechanisms of ecDNA

### ecDNA propels high oncogene expression because of high copy number and untied chromatin

Through the analysis of allele-specific RNA sequencing, it was found that ecDNA can be used as a template for gene transcription 11. More intriguingly, oncogenes encoded on ecDNA generally have high expression levels. The abundance of ecDNA-derived transcripts was the highest in tumours 11. Generally, ecDNA facilitates oncogene overexpression in two modes. First, ecDNA with a high copy number level was detected in tumours, where its number can reach the hundreds 11. The ability of ecDNA to embrace a high copy number is probably related to the uneven separation of ecDNA. Although ecDNA segregates unevenly, ecDNA is not lost during mitosis, such as moving into micronuclei. EcDNAs might tether themselves to chromosomes during cell division to avoid the loss. In fact, a high copy number is only one explanation for the high expression of oncogenes. Second, ecDNA containing highly accessible chromatin has been confirmed by assays of accessible chromatin (ATAC-seq, ATAC-see, etc.) **(Figure 4B)**. Given that the circular topological structure endows ecDNAs with higher chromatin accessibility, ecDNA boasts higher transcriptional activity. Consistently, recent studies have reported that even chromosomal DNA and ecDNA have similar copy numbers, ecDNA exhibits a huge advantage in transcribing oncogenes 11, 53.

### Cis-regulation function of ecDNA

The information encoded in DNA is usually determined by its physical appearance. As long as the DNA forms a circular structure, the fragments of ecDNA will develop a novel chromatin domain that is different from their linear chromosome structure. Interestingly, because DNA forms a circular conformation, it is possible to bring distant DNA elements (enhancers) nearby, forming a new cis-regulatory configuration that is impossible for chromosomal DNA 11. Thus, ecDNA acts as a stronger enhancer hijacking vector to participate in the evolution of tumours 54.

Enhancer hijacking that occurs on ecDNA typically exhibits two patterns: the local enhancer hijacking model and the distal enhancer hijacking model. In the local enhancer hijacking model, the ecDNA circularizes and hijacks the enhancers at the distal end of the oncogene to bring them into the vicinity. Insulators insulate enhancers from oncogenes, which can prevent enhancers from participating in oncogene regulation in the chromosomal DNA **(Figure 4C, top)**. As long as the enhancers and the oncogene coexist in an ecDNA circular domain, the enhancers can cross the insulator and participate in the transcriptional regulation of the oncogene **(Figure 4C, bottom)**. It has been found that the oncogene EGFR often co-amplifies with upstream enhancers and forms ecDNA in glioblastoma, generating new enhancer-oncogene contacts and promoting tumour progression 26. If we intervene in the ecDNA-specific domain, it is possible to weaken the transcriptional regulatory function of the enhancer, thereby affecting oncogene expression and delaying tumour progression.

The distal enhancer hijacking refers to the process in which distant enhancers and oncogene fragments join together to form ecDNA loops **(Figure 4D)**. These DNA segments containing enhancers may originate from identical or dissimilar chromosomes. Thus, distal enhancer hijacking creates a unique domain on ecDNA that complicates oncogene regulatory mechanisms on ecDNA. EcDNA-mediated remote enhancer hijacking is prevalent in neuroblastoma, which often leads to a poor prognosis 27, 28.

Additionally, we believe that ecDNA could employ epigenomic factors to gain survival advantages. EGFR ecDNA formation relies on H3K9 methylation 55. Recently, Zhang et al. revealed that Lysine demethylase 5B (KDM5B) recruits SET domain bifurcated histone lysine methyltransferase 1 (SETDB1) to repress the transcription of transposable elements (TEs) by H3K9me3 modification, and thereby inactivating cGAS-STING pathway 56. We think, the effect of KDM5B on ecDNA needs to be viewed from both positive and negative aspects. On the one hand, high levels of KDM5B might deplete SETDB1, leading to reduced ecDNA formation. On the other hand, ecDNA can enhance the immunogenicity of tumor cells through the cGAS-STING pathway, but it still promotes cancer progression. We speculate that KDM5B-mediated immunogenic blockade may provide a survival advantage to ecDNA-harboring tumor cells.

### ecDNA acts as a movable trans-acting element

DNA is wrapped tightly around histones and packed into a linear chromosomal structure. Different chromosomes occupy specific spaces within the nucleus, which forms chromosomal domains. This spatial structural domain is significant for the function and physical stability of chromosomes, and can also limit the interactions of chromosomes 57, 58. It is unknown whether ecDNA has a spatial domain, but ecDNA is certainly scattered within the nucleus. Moreover, ecDNA can interact with other chromosomes owing to its preponderance of high copy number, small physical size, and mobility.

A later study regarding interactions between ecDNA and chromosomal DNA indicated that the function of ecDNA equates to a movable trans-acting element **(Figure 4E)**. Interactions between ecDNA and chromosomal DNA appear to be genome-wide. Moreover, up to a few hundred trans interaction sites, where ecDNA interacts with chromosomal DNA, were illuminated by RNA polymerase II ChIA-PET (chromatin interaction analysis with paired-end tag sequencing), which further confirmed that their interactions contribute to higher transcriptional activity. Furthermore, ecDNA contains a large number of enhancers, and these enhancers often interact with chromosomes. From what has been discussed above, ecDNA is involved in regulating the expression of chromosomal genes, especially oncogenes, owing to the characteristic of its mobile enhancer 29. Gen et al. found that miR-766-5p can reduce levels of H3K27ac at MYC super-enhancers via CBP and BRD4 suppression 59. Zhu et al. reported that ecDNAs can act as mobile super-enhancers, thereby driving genome-wide transcription 29. We believe that since microRNA can inhibit the activity of H3K27ac on linear chromatin, it should also reduce the level of H3K27ac on ecDNAs and inhibit the biological activity of ecDNAs as super-enhancers. However, the interaction between microRNA and ecDNA is rarely reported. If the molecular mechanism of action between the two can be elucidated, it will open up a new field for cancer research.

Due to the high level of amplification and mobility of ecDNA, the interaction between ecDNA elements is expected **(Figure 4F)**. Recent studies have shown that ecDNA tends to physically aggregate, and ecDNA elements can gather to form ecDNA hubs **(Figure 4F)**, which can promote the expression of oncogenes. For instance, ecDNAs containing MYC-PVT1 without enhancers can hijack the enhancers from other ecDNAs within the ecDNA hub to enhance the expression of the fusion gene 60.

### ecDNA drives somatic rearrangement

**Figure 5:**
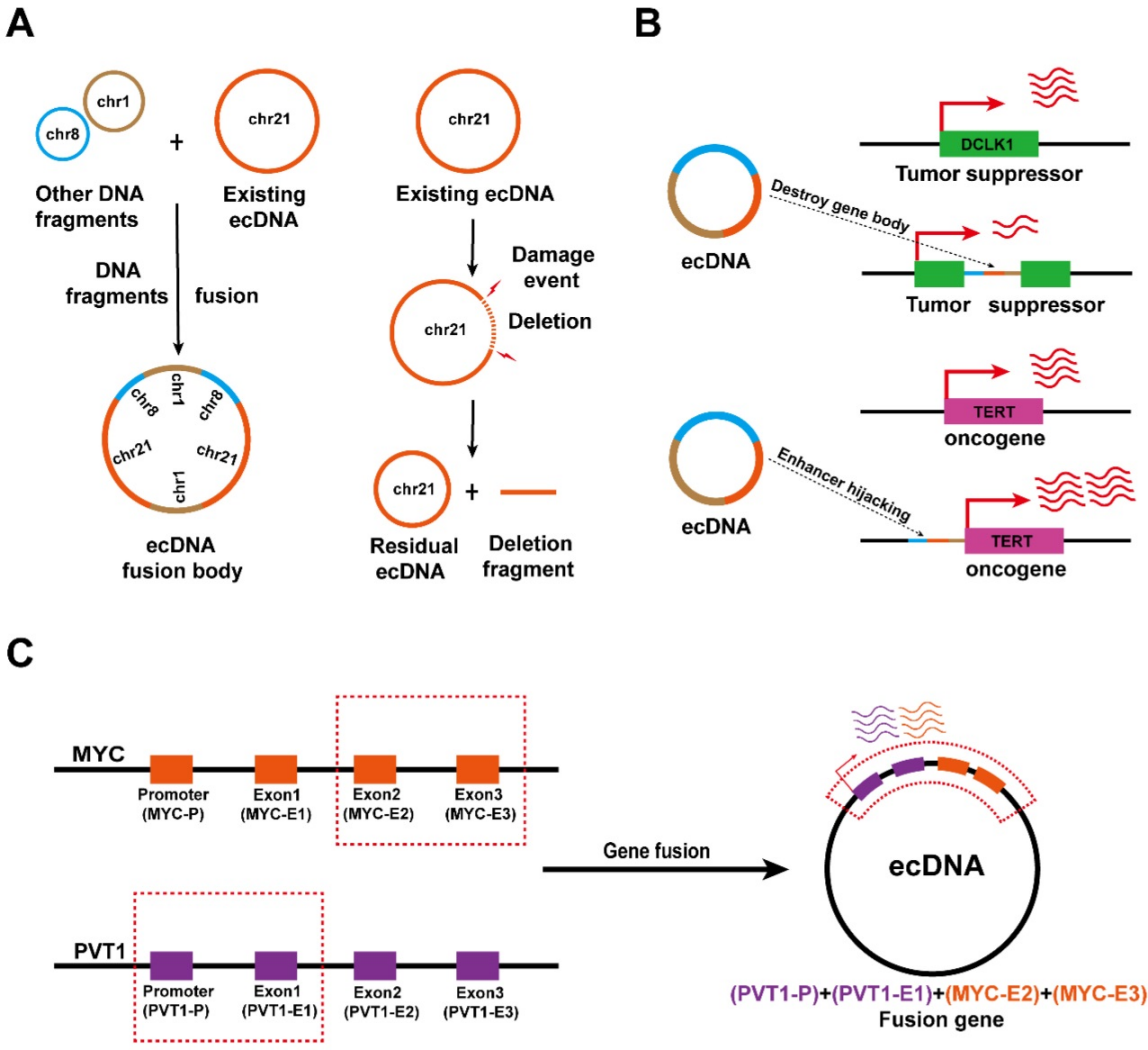
**ecDNA drives somatic rearrangement (A)** ecDNA fusion and deletion: DNA fragments can be added into or deleted from an existing ecDNA, creating new ecDNA types in cancer. **(B)** ecDNA reintegration: ecDNA can reintegrate into the coding region of tumour suppressor genes, thereby downregulating gene expression in the integration site (top). EcDNA can reintegrate into the vicinity of the oncogene promoter to enhance oncogene transcription (bottom). **(C)** Gene fusion on ecDNA: ecDNA may enable gene fusion because it is formed by the circularization of DNA fragments from one or more chromosomes.

Now, the concepts towards the instability of ecDNA can be extended to the changes in physical conformation and spatial dynamics, where ecDNA may alter their sequence and localization in the nucleus 37, 61, 62. The analysis of ecDNA structure suggests that the novel ecDNA biogenesis can be attributed to the addition of new DNA fragments or the deletion of original DNA fragments in the original ecDNA in cancer 63, 64. For example, in small cell lung cancer cell line GLC1, the ecDNA carrying the sequences of chromosomes 1, 8 and 21, respectively, will undergo sequence rearrangement to form a large ecDNA loop **(Figure 5A, left)**, and damage events of existing ecDNA can also lead to the deletion of a certain sequence **(Figure 5A, right)** 63. There are extensive interactions between ecDNA and chromosomal DNA 29, 60. Therefore, the functional units of ecDNA rearrangement may be related to these ecDNA interaction foci.

Early observations of Southern blotting and contemporary sequencing techniques combined with bioinformatics analysis found that some ecDNAs are able to aggregate and subsequently reintegrate into chromosomal DNA 14, 37, 62. EcDNA fragments that are integrated into chromosomal DNA disrupt the integrity of the gene and favor oncogene expression at integration sites. It was found that ecDNA reintegration can disrupt the functional integrity of the tumour suppressor gene DCLK1, thereby downregulating DCLK1 expression **(Figure 5B, top)**, and can also act as a hijacked enhancer to regulate TERT expression in neuroblastoma **(Figure 5B, bottom)** 27, 65.

Interestingly, since the fragments forming ecDNA may come from different chromosomes, ecDNA biogenesis is also accompanied by gene fusion 60, 64, 65. Recent studies have confirmed the presence of the PVT1-MYC fusion gene in tumour ecDNA, thus the PVT1 promoter can also enhance the transcription of MYC **(Figure 5C)** 53. Nevertheless, the effects of ecDNA-mediated gene fusion in tumours need to be further explored.

## ecDNA in cancers

**Figure 6:**
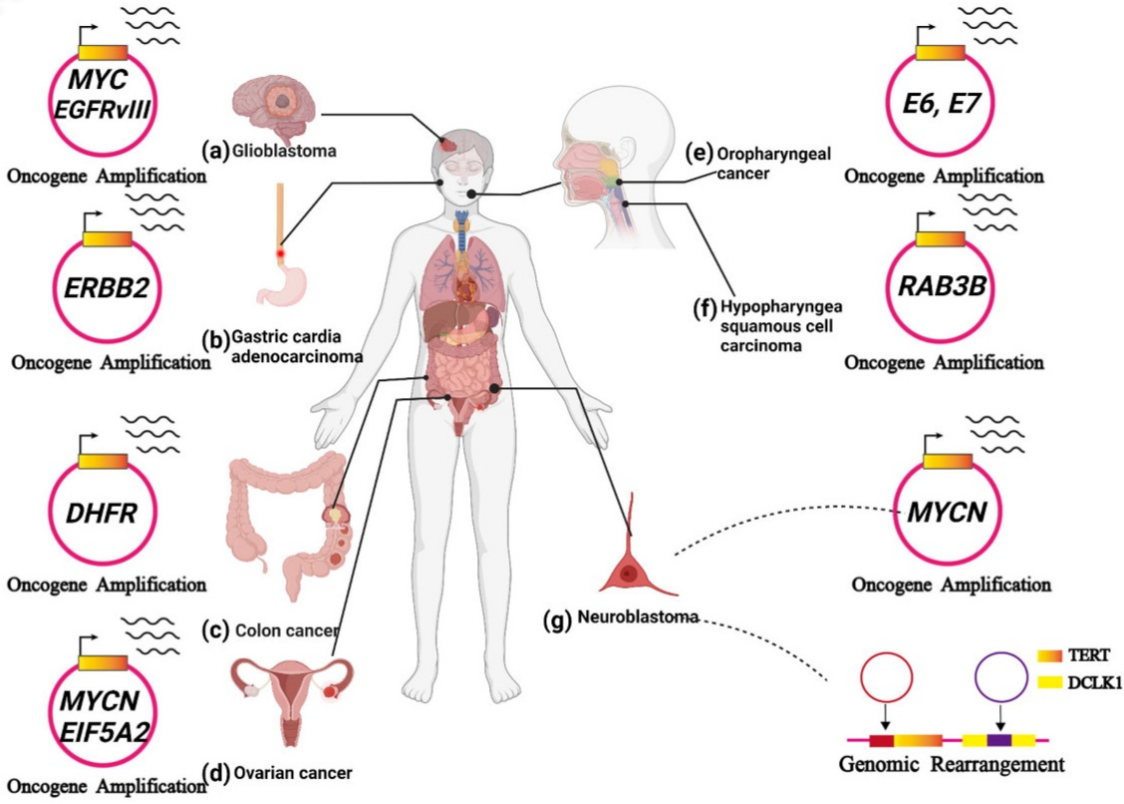
**ecDNA is related to tumour progression. (A)** In glioblastoma, ecDNA initiates a great number of carcinogenic amplifications and mutations. **(B)** In gastric cardia adenocarcinoma, ecDNA-derived ERBB2 focal amplifications might serve as a prognostic biomarker. **(C)** In colon cancer, ecDNA-mediated gene amplification accounts for drug resistance. **(D)** In ovarian cancer, noncoding regions (MARs) on ecDNAs could enhance the expression of oncogenes near the MARs, including MYCN and EIF5A2.** (E)** In HPV-mediated oropharyngeal cancer: human-viral hybrid ecDNA could prompt oncogene expression and tumour evolution. **(F)** In hypopharyngeal squamous cell carcinoma, the RAB3B gene was amplified on ecDNA, and RAB3B protein could induce a drug-resistant phenotype by promoting autophagy.** (G)** In neuroblastoma, ecDNA involves the amplification and rearrangement that contribute to tumorigenesis.

ecDNA has been observed in a variety of cancers and plays a key role in tumour progression. In this section, we present recent reports exploring our understanding of ecDNA's biochemical functions** (Figure 6A-G).**

### ecDNA in glioblastoma

ecDNA initiates a great number of carcinogenic amplifications and mutations and has been identified in 10-40% of glioblastomas^66^, ^67^. In glioblastoma, a variety of oncogenes were amplified on ecDNA, such as EGFR, MYC, CDK4, MDM2 and PDGFRA **(Figure 6A)** 66, 68**.** deCarvalho et al. demonstrated that both ecDNA-mediated oncogene amplification and somatic single-nucleotide variants are involved in the dynamic evolution of glioblastoma 25.

The amplification of the epidermal growth factor receptor (EGFR) gene through double minutes is frequently observed in glioblastoma 39. Zhou et al. observed increased invasiveness, heterogeneity, and radioresistance in GBM cell lines containing EGFR-encoding double minutes. However, it is unclear whether eliminating EGFR-encoded double minutes to downregulate the expression of EGFR alleviates these malignant phenotypes 69.

In glioblastoma, EGFR is frequently mutated, forming the oncogenic variant EGFRvIII. EGFRvIII prompts tumour growth but makes glioblastoma cells more sensitive to EGFR tyrosine kinase inhibitors (TKIs) 70. Nathanson et al. found that resistance to EGFR tyrosine kinase inhibitors (TKIs) occurs after the elimination of EGFRvIII from ecDNA. After inhibitor withdrawal, the reemergence of EGFRvIII on ecDNA follows. They then observed EGFRvIII gene amplification on ecDNA **(Figure 6A).** Thus, oncogenes amplified on ecDNA might also serve as predictive biomarkers for therapies 24.

Subsequently, a new type of mutation in cancer, termed amplification-linked extrachromosomal mutations (ALEMs), was proposed. Oncogenic focal amplification of some oncogenes (such as EGFR and PDGFRA) on ecDNA may increase the chance of functional mutations. Amplification-linked extrachromosomal mutations (ALEMs) are common in glioblastoma and low-grade gliomas, as well as other tumours. The ecDNA-mediated ALEMs explain the amplification of mutated oncogenes, including EGFR and PDGFRA, in glioblastoma 24. No doubt, subsequent research should focus on developing sequencing based detection tools that can effectively identify ecDNA and better understand how it is formed. If we can block these mechanisms, we can prevent the evolution and even the biogenesis of glioblastoma.

### ecDNA in gastric cardia adenocarcinoma

Oncogenic focal amplification plays a pivotal role in the progression of gastric cardia adenocarcinoma and is associated with poor prognosis 71. Recently, Zhao et al. identified a mass of ecDNAs in Chinese gastric cardia adenocarcinoma patient samples, and a variety of amplified oncogenes were observed on them, including ERBB2, EGFR, and CCNE1 **(Figure 6B)**. Moreover, they explored the correlations between the focal amplifications (including ecDNA-derived circular amplicons) and prognosis based on an immunohistochemistry analysis from 1,688 gastric cardia adenocarcinoma patients.

The results show that ERBB2-positive patients have worse prognosis than ERBB2-negative patients when their survival time is less than two years. Notably, if the patients' survival time is longer than two years, the tendency could be completely reversed. Therefore, ecDNA-derived ERBB2 focal amplifications might serve as a favourable prognostic biomarker in gastric cardia adenocarcinoma patients 72.

As mentioned above, further analysis of ecDNA in GCA progression is quite meaningful and may provide several more sensitive biomarkers and effective targets for therapies.

### ecDNA in colon cancer

As one of the oncogenic genomic features, gene amplification can markedly prompt tumour evolution and drug resistance 73. MTX, an inhibitor of dihydrofolate reductase (DHFR), plays an antitumor role in a variety of cancers by interfering with the synthesis of cellular DNA. However, colon cancer often develops resistance to MTX due to DHFR gene amplification 74. Treating HT29 cells with MTX significantly increased DHFR gene expression via ecDNA-mediated amplification **(Figure 6C)**. Additionally, withdrawing MTX treatment decreased ecDNA-mediated DHFR amplification in MTX-resistant cells. The loss of the DHFR amplicon in MTX-resistant cells can suppress their capacity to generate resistance. As expected, when these MTX-resistant cells that lost the DHFR amplicon were re-exposed to MTX, the cells may become responsive to the second round of MTX treatment. These observations provide a promising treatment strategy for overcoming drug resistance induced by ecDNA-mediated amplification 75. In 2015, Meng et al. revealed the importance of nonhomologous end joining (NHEJ) in ecDNA formation. They found that depleting DNA-PKc (an NHEJ-related protein) reduces ecDNA-mediated DHFR amplification and increases MTX sensitivity. Accordingly, NHEJ is presented as a promising target for overcoming MTX-resistant colon cancers 76. They also demonstrated that homologous recombination activity was upregulated in MTX-resistant cells. In their study, the silencing of the BRCA1 gene (a major player in homologous recombination) decreased the amount of ecDNA and downregulated the expression of ecDNA-amplified oncogenes. Furthermore, silencing BRCA1 makes MTX-resistant cells containing ecDNA more sensitive to MTX but has no discernible effect on MTX-resistant cells containing HSRs. Therefore, the homologous recombination pathway may also serve as a target to advance current therapies by decreasing ecDNA-mediated oncogenic amplification 77. Targeting ecDNA-mediated drug resistance gene amplification may shed new light on the treatment of colon cancer, further advancing current therapies and improving patient outcomes and survival.

### ecDNA in ovarian cancer

Jin et al. found that noncoding regions on ecDNAs perform a considerable function in regulating gene expression. They discovered several matrix attachment regions (MARs) within an ecDNA derived from UACC-1598 cell line using sequence analysis and bioinformatics analysis. Moreover, they have identified the interaction between the MARs and the nuclear matrix, which results in a significant enhancement of gene expression. Transfecting the MAR construct into 293 T cells could also enhance the expression of oncogenes located near the MARs, including MYCN and EIF5A2 **(Figure 6D)** 78. Accordingly, ecDNAs might play an important role in the regulation of gene expression in ovarian cancer.

Raymond et al. performed a clinical trial to evaluate the efficacy of hydroxyurea on inhibiting double minutes in cancer cells from patients with ovarian cancer. They demonstrated that low-dose hydroxyurea could decrease the number of double minutes in cancer cells 79. However, clinical evaluation of the strategy does not induce tumour shrinkage as expected.

Although the role of ecDNA in ovarian cancer is not thoroughly understood, further research is warranted. On the one hand, noncoding regions of ecDNA can cause increased oncogene expression, which may provide a new target for ovarian cancer treatment 78. On the other hand, exploring more drugs that can prompt the elimination of ecDNA in tumour cells is meaningful.

### ecDNA in HPV-mediated oropharyngeal cancer

Previous reports have theorized that hybrid human-virus ecDNA formation could be a potential mechanism for increased expression of the HPV oncogenes E6 and E7 **(Figure 6E)** 80-84.

Later, Deshpande et al. demonstrated that the HPV-mediated oropharyngeal cancer cell line UPCI: SCC090 contains hybrid human-viral ecDNA 85. Recently, Pang et al. revealed that ecDNA was present in nearly all HPVOPCs. Additionally, a novel human-viral hybrid ecDNA was also identified in HPVOPC 86. Moreover, hybrid ecDNA highly expresses fusion transcripts that contain promoter and oncogene sequences of HPV. These fusion transcripts are associated with downstream human transcripts. These downstream transcripts could drive carcinogenesis and immune evasion 86. Viral promoters and genes account for the high expression of hybrid transcripts and human oncogenes in hybrid ecDNA structures, which is facilitated by high levels of chromatin modifications (such as H3K27 ac) in ecDNA 11, 86.

Human-viral hybrid ecDNA is an exclusive feature of HPVOPC. In this way, the hybrid ecDNA boasts an uneven inheritance, thereby driving rapid tumour evolution even in the face of targeted therapies. Thus, the characterization of ecDNA in oropharyngeal cancers will provide some prognostic biomarkers for clinical treatment. Additionally, clarifying the role of ecDNA in HPVOPC will contribute to the development of HPVOPC therapy.

### ecDNA in hypopharyngeal squamous cell carcinoma

Cisplatin resistance leads to the malignant progression of hypopharyngeal squamous cell carcinoma (HSCC). Recently, Lin et al. found that the gene encoding RAB3B might be amplified on ecDNA **(Figure 6F)**. Moreover, they demonstrated that RAB3B protein could induce a cisplatin resistance phenotype in HSCC by promoting autophagy. However, they did not verify that the sequences of the RAB3B genes on ecDNA can be directly transcribed. ecDNA-mediated drug resistance-related gene amplification might explain cisplatin resistance in hypopharyngeal squamous cell carcinoma (HSCC) 87. Further study is required to clarify the undiscovered aspects of ecDNA in hypopharyngeal squamous cell carcinoma (HSCC).

### ecDNA in neuroblastoma

The first appearance of ecDNA was observed in the metaphase of neuroblastoma cells in 1965 12. Later, Alt et al. confirmed the presence of a new oncogene, MYCN, in ecDNA in the neuroblastoma cell line. This was the first report that confirmed that oncogenes were located on ecDNA 19. MYCN amplification drives one in five cases of neuroblastoma, and the MYCN gene is mainly amplified on ecDNA and HSRs **(Figure 6G)** 14. Recently, Helmsauer et al. examined the structure of MYCN amplicons in neuroblastoma ecDNA and revealed the mechanism of ecDNA-mediated MYCN amplification. There are two main aspects to the mechanism: 1) local enhancer-induced MYCN amplification in neuroblastoma ecDNA. 2) Distal enhancer-induced MYCN amplification in neuroblastoma ecDNA 28. These findings may provide promising therapeutic targets for MYCN-amplified tumours.

Recently, it has been reported that ecDNA can drive oncogenic genome rearrangement in neuroblastoma 27, 34. Genome rearrangement usually causes abnormal gene expression and mutation. Previous studies have shown that integrating adjacent ecDNA fragments into the oncogene TERT increases TERT expression. In contrast, integrating ecDNA fragments into the tumour suppressor DCLK1 leads to a lower level of DCLK1 expression **(Figure 6G)** 27. Later, using whole-genome sequencing data of neuroblastoma, Rosswog et al. identified a novel genome rearrangement that drives oncogene amplification in many human malignancies, especially neuroblastoma. The novel rearrangement involves chromothripsis and circular recombination and eventually contributes to the evolution of seismic amplification 34. It is worth noting that Koche et al. hypothesized that ecDNA-derived genome rearrangement could result in mutagenic processes in neuroblastoma, which have functional consequences beyond the amplification of oncogenes 27.

The abovementioned molecular signatures are being evaluated to better understand the disease, which would help identify novel targets and improve the treatment outcomes of patients with neuroblastoma.

## Advancing ecDNA toolbox

With the rapid development of the ecDNA field, robust tools are continuously emerging. Here, we will present current effective tools for ecDNA research.

### High-throughput tools

Sequencing and analysis tools can be attractive solutions to assessing some basic properties of ecDNA from publicly accessible cancer genome databases and patient-derived tumour tissue. Here, we present some effective sequencing-based approaches for ecDNA.

#### Circle-seq

**Figure 7:**
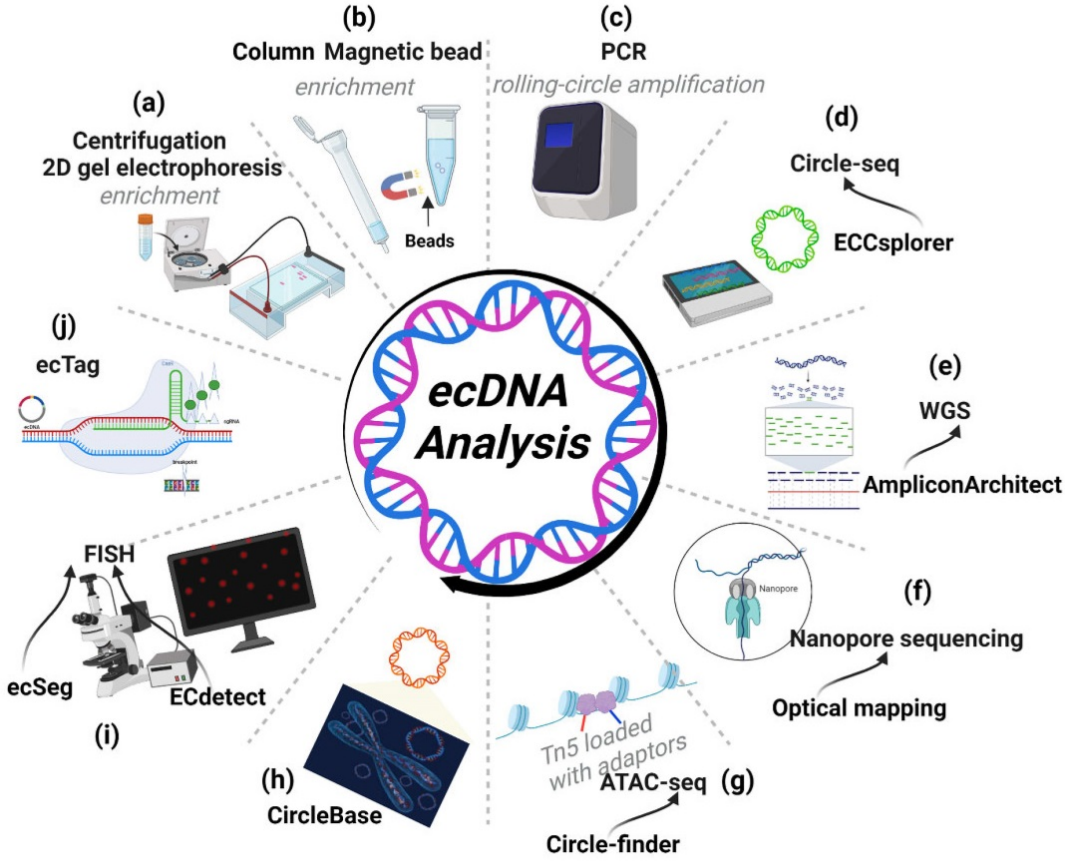
**Tools for ecDNA research. (A-D)** Workflow of Circle-seq: Circle-seq is a specialized sequencing method for ecDNA requiring enrichment protocols before sequencing. Enrichment protocols include density gradient centrifugation, 2D gel electrophoresis (A), column- or magnetic bead-based enrichment methods (B) and rolling-circle amplification (C) of ecDNA before sequencing. (D) Following circle-seq, ECCsplorer enables convenient and efficient discovery of potential ecDNA. **(E)** WGS: AmpliconArchitect can extract ecDNA information from WGS data. **(F)** Nanopore sequencing: nanopore long-read sequencing and optical mapping can identify the complex architecture of ecDNA. **(G)** ATAC-seq: ATAC-seq with Circle-finder software could also identify ecDNA from cancer tissues. **(H)** CircleBase: CircleBase is a platform for integrating and analysing ecDNA resources, thereby screening for functional ecDNAs and interpreting their molecular mechanisms. **(I)** FISH: FISH can identify the genes amplified on ecDNA. ECdetect is a new integrative analysis pipeline that can quantify ecDNA from metaphase cells stained with DNA stains (DAPI). ecSeg can ameliorate the sensitivity of detecting DAPI-stained ecDNAs in metaphase cells by employing a deep neural network. **(J)** ecTag: ecTag can label ecDNA with multiple fluorescent molecules in living cells.

Circle-seq is a specialized sequencing method for ecDNA 9, 88. Searching for ecDNAs by circle-seq requires enrichment protocols before sequencing. Traditionally, ecDNA isolation involves two steps: 1) caesium chloride-ethidium bromide density gradient centrifugation and 2) 2D gel electrophoresis **(Figure 7A)** 4, 89**.** Modern ecDNA isolation involves two new steps: 1) column- or magnetic bead-based methods to isolate high-molecular-weight DNA and 2) removing linear DNA by exonuclease **(Figure 7B)**^90^**.** Additionally, the enrichment protocols require rolling-circle amplification of ecDNA before sequencing** (Figure 7C).** In neuroblastoma, circle-seq has revealed the landscape of MYCN amplicons, which are amplified on ecDNA 27.

Recently, Mann et al. developed a bioinformatics pipeline named ECCsplorer (https://github.com/crimBubble/ECCsplorer). Following circle-seq, ECCsplorer enables convenient and efficient discovery of potential ecDNA** (Figure 7D)** 91**.**

In comparison to traditional sequencing techniques, prepared enrichment theoretically improves the efficacy of identifying ecDNA by removing other amplicons. Nevertheless, there is still a technical challenge in protecting ecDNAs from shearing by exonuclease, especially in the case of large ecDNAs with several megabases. Theoretically, it is possible to improve sample integrity during the enrichment stage through the use of technologies such as automatic liquid handling. During the rolling-circle amplification of ecDNA, Phi29 polymerase demonstrates high fidelity, but amplification bias and the possibility of mutations occurring during ecDNA enrichment need to be considered 92.

#### WGS

Whole-genome sequencing (WGS) can provide access to all information about the genome in an organism. Searching for discordant reads is a viable method for identifying ecDNA. In general, 10× WGS coverage is adequate to detect ecDNA 53. Additionally, AmpliconArchitect software can be effective in analysing amplicons and extracting ecDNA information from WGS data 85. Employing discordant read mapping, AmpliconArchitect can generate an amplicon graph that represents the architecture (order and orientation) of excised DNA segments. Employing AmpliconArchitect to predict ecDNA shows a favourable performance (85% positive rate and 83% sensitivity). Comparing its prediction to metaphase FISH in cancer cells, the accuracy of AmpliconArchitect was further confirmed 11, 53. Moreover, Kim et al. used WGS data from 3,212 cancer patients in the TCGA and ICGC (International Cancer Genome Consortium) cohorts to explore the ecDNA landscape with the help of AmpliconArchitect 53. Notably, the results from WGS using AmpliconArchitect are almost entirely consistent with those obtained from Circle-Seq, a method aimed at detecting ecDNA, further supporting the fidelity of AmpliconArchitect** (Figure 7E)** 72**.**

#### Nanopore sequencing

The main problem with WGS, however, is that its read length is relatively short (100 to 200 bp); it cannot identify the complex structural rearrangements of amplicons as large as ecDNA, which is often between 1-3 megabases in length. Technologies such as nanopore long-read sequencing and optical mapping are proven methods to resolve the ecDNA structure and can mitigate this limitation **(Figure 7F)** 11, 28**.** The combination of WGS and optical mapping, in conjunction with AmpliconReconstructor, a recently developed algorithm, is proven to be an extremely accurate and economical technique for resolving complicated amplicon architectures 93.

#### ATAC-seq

Other sequencing and analysis tools, such as ATAC-seq with Circle-finder software, could also identify ecDNA from cancer tissues** (Figure 7G)** 94**.** In addition, sequenced genomic fragments usually do not contain exclusive junction sequences, thus making it impossible to differentiate the genomic segments from different ecDNA. To improve our efficiency for identifying ecDNA by sequencing, a side-by-side comparison of WGS and ATAC-seq followed by their respective software analysis is necessary.

### CircleBase: a platform for integrating and analysing ecDNA resources

Zhao et al. have developed a new platform, CircleBase (http://circlebase.maolab.org), that integrates and analyses human ecDNAs from accessible public data. Moreover, it can predict the regulatory networks among ecDNAs by integrating and analysing relevant databases. CircleBase boasts several advantages. (i) CircleBase enables visualization of the functions and annotations of ecDNAs; (ii) CircleBase is equipped with a ranking system (based on the Gaussian distribution model) for ecDNA and (iii) provides overall ecDNA annotations **(Figure 7H)** 95**.** Thus, CircleBase is a robust tool for explaining the functions and mechanisms of ecDNA and will facilitate the exploration of tumour heterogeneity and genome diversity.

### Imaging-Based Approaches

To see is to believe. The gold standard to determine the presence of ecDNA is to capture its microscopic images. First, the preparation of cells at metaphase is needed. A hypotonic buffer is used to swell mitotic cells. Next, the swollen mitotic cells are fixed and stained with DNA stains to label chromosomes and ecDNA. Finally, with downstream FISH, we can definitively identify the gene amplified on ecDNA. Moreover, we can also directly obtain images of ecDNA by fluorescence microscopy.

Recent developments in algorithms and software have enabled the quantitative determination of ecDNA across large cell populations. Turner et al. developed a new integrative analysis pipeline named ECdetect, which quantifies ecDNA from metaphase cells stained with DNA stains (DAPI). Although it provides very high precision, it achieves a slightly lower sensitivity than visual counting and thus quantifies less ecDNA, especially ecDNA particles near chromosomes 53. ecSeg is also an effective tool. Employing a deep neural network, ecSeg can ameliorate the relatively low sensitivity of detecting DAPI-stained ecDNAs in metaphase cells** (Figure 7I)** 96**.** However, the above two algorithmic models were developed primarily by training on images of cancer cell lines from one team. Thus, additional training data generated by other teams with different sample types are required to optimize the abovementioned analysis tools.

Advances in biological techniques have also greatly improved the detection of ecDNA. Recently, Yi et al. developed a CRISPR-based ecDNA tracking system called 'ecTag'. Leveraging DNA junction sites, ecTag can label ecDNA with multiple fluorescent molecules in living cells **(Figure 7J).** With the help of ecTag, the spatial and temporal dynamics of ecDNA can be effectively captured. Moreover, the hypothesis that uneven segregation of ecDNA could contribute to intratumoral heterogeneity has been further confirmed by ecTag 97.

Nevertheless, imaging-based technology has some limitations. Occasionally, it is not feasible to prepare metaphase cells. FISH cannot be performed without sequence information for the ecDNA. In addition, the detection throughput of the imaging approach is low. Consequently, well-suited, sensitive, and high-throughput tools are needed.

## The role of ecDNA in cancer treatment

**Figure 8:**
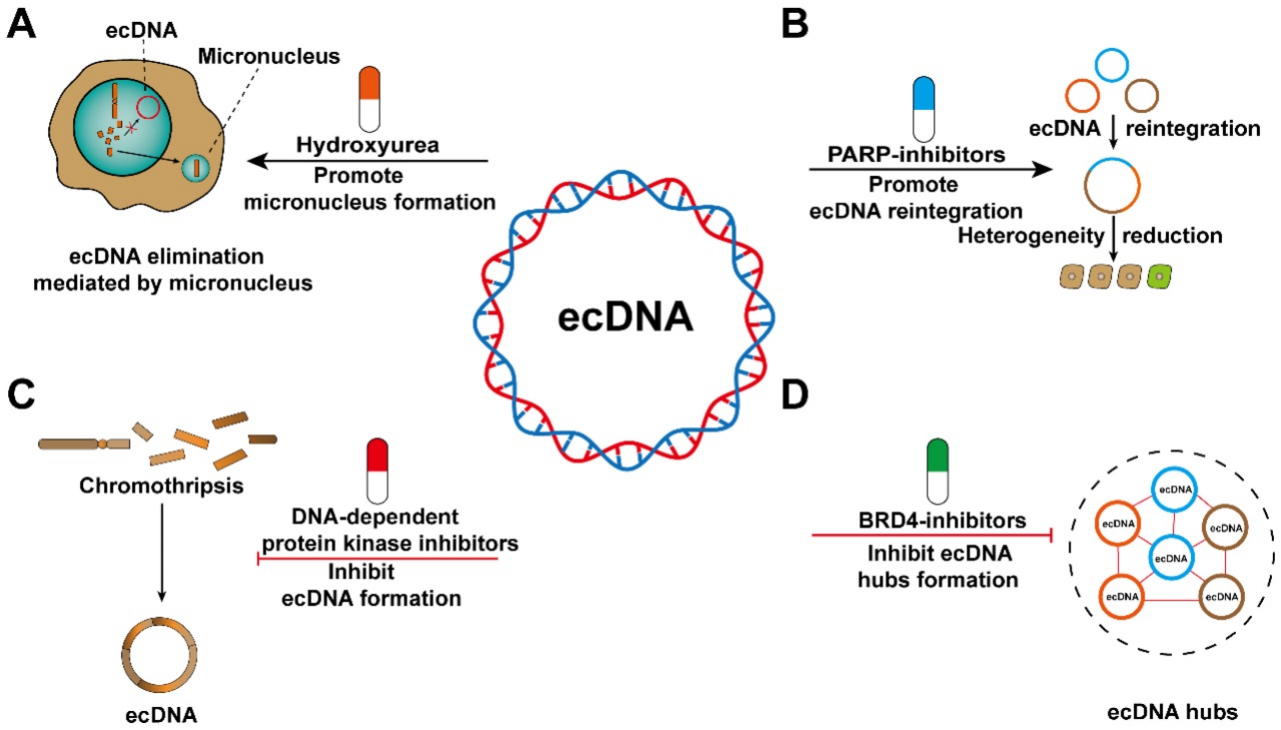
** Potential therapies that target ecDNA. (A)** ecDNA can be eliminated through micronuclei. Hydroxyurea (HU) increases the frequency of micronucleus formation. **(B)** ecDNA reintegration might decrease the heterogeneity of cancer cells. PARP inhibitors can increase the frequency of ecDNA reintegration. **(C)** Chromothripsis can result in ecDNA formation. DNA-dependent protein kinase inhibitors will decrease the amount of ecDNA formed during chromothripsis events. **(D)** ecDNA hubs can prompt intermolecular regulation among ecDNAs and drive oncogene expression. BRD4 inhibitors can inhibit the formation of ecDNA hubs.

Cancer cells gain survival advantages by continually altering their genomes. Oncogene amplification is a classic form of genome alterations. Cancer cells are addicted to ecDNA because ecDNA is a carrier that maintains oncogene amplification 11, 26, 98. Therefore, limiting the survival of cancer cells by eliminating ecDNA may be an effective therapeutic approach 99-103. However, there are currently few targeted drugs related to ecDNA 24, 79. Most notably, since the formation of micronuclei contributes to ecDNA elimination, it has been confirmed that the antimetabolite hydroxyurea (HU) can enhance this process. It has been proven that HU does not show good clinical antitumor activity, so HU is not used in the treatment of ecDNA-positive tumours 79. Although HU did not achieve the desired effect, existing observations might provide the research basis for further drug screening **(Figure 8A)**. Additionally, we believe that the ecTag might uncover the mechanisms contributing to ecDNA elimination by tracing the spatiotemporal dynamics of ecDNA moving into micronuclei.

Tumour heterogeneity is an important cause of drug resistance, and the increased frequency of ecDNA reintegration may reduce heterogeneity among cancer cells. Recently, PARP has been shown to reduce the frequency of ecDNA reintegration and thus may be a candidate therapeutic target 23. However, ecDNA reintegration also inevitably exerts some adverse effects, such as affecting the expression of adjacent oncogenes 23, increasing genomic instability 104, and destroying the sequence structure of tumour suppressor genes 27. Therefore, further study is required to avoid the side effects of ecDNA as much as possible to improve clinical therapies **(Figure 8B)**.

Reducing the risk of ecDNA biogenesis might serve as a promising treatment for cancer patients. Nonhomologous end joining during DNA damage repair is involved in ecDNA generation, and DNA-dependent protein kinase inhibitors can interfere with this process. The emergence of ecDNA is significantly reduced after treatment with the drug 23. EcDNA is often found in tumour samples from patients with different cancer types 53. Unfortunately, there is a lack of biomarkers that can detect the biogenesis of ecDNA in cancer patients **(Figure 8C)**.

The spatially abnormal distribution of ecDNA, which eventually forms ecDNA hubs, can cause trans-interactions between ecDNA and between ecDNA and chromosomal DNA. Therefore, the spatially abnormal distribution of ecDNA may represent a treatment-related vulnerability. Proteins involved in the formation of ecDNA hubs have been considered as emerging potential therapeutic targets 60. The stability of ecDNA hubs is inseparable from the existence of the extraterminal domain (BET) protein BRD4 105-107. However, further research is needed to determine whether BRD4 plays a decisive role in maintaining ecDNA hubs stability **(Figure 8D)**. If we want to treat cancer by interfering with the formation of ecDNA hubs, there are still some aspects that need to be elucidated. For example, are the segregation of ecDNA in the form of singletons or smaller hubs? Whether the composition of ecDNA hubs changes with cell passage? Whether the spatial distribution of ecDNA hubs in the nucleus is random or directed? Are there differences in the composition of ecDNA hubs in different cancer species?

Given the large number of new research results on the occurrence, maintenance and function of ecDNA, it is feasible to develop related drugs targeting ecDNA. In the future, therapeutic targets of ecDNA may develop as a new approach to conquer tumours.

## Conclusion and future perspectives

ecDNAs are circular DNA that contain oncogenes and are responsible for tumorigenesis. Numerous mechanisms have been proposed to clarify the genesis of ecDNA, including chromothripsis, slight DNA damage and relegation, fork stalling and template switching, and genome instability. However, further studies are required to reveal the mechanisms by which these ecDNAs are formed. ecDNAs exhibit unique structure and function which enable them to accelerate the evolution of tumours. ecDNAs contribute to oncogene overexpression and somatic rearrangement, ultimately causing cancer progression and tumour heterogeneity. These processes eventually lead to cancer cells becoming resistant to various stresses and therapies. A series of pioneering tools for ecDNA research have been developed in recent years. For example, the development of “ecTag” has removed the barriers in exploring the temporal dimension of ecDNA. Further understanding of ecDNA requires continuous exploration and advancement of technology.

The field of ecDNA is rapidly advancing. Currently, there are still some key areas that must be addressed. The first is clarifying the basic biology of ecDNA. It is necessary to detail the mechanisms of chromosomal instability in ecDNA formation and further explore the roles of NHEJ pathways and double-strand breaks in this process. Furthermore, the influence of ecDNA reintegration on genome rearrangement and its relocation as HSR is still not fully understood. It has been reported that HSR formation is associated with drug resistance, but the detailed mechanisms remain largely unknown.

Second, ecDNA is correlated with tumour evolution. In the future, unremitting explorations will be necessary to better understand the molecular pathogenesis mechanisms and function of ecDNA in tumours. Moreover, a recent study demonstrated that ecDNA can activate innate immune signalling pathways such as cGAS-STING. Thus, the role of ecDNA in the tumour immune microenvironment deserves more attention.

Third, as we deepen our understanding of ecDNA, we will be able to overcome ecDNA-associated clinical pathogenesis. Due to their relative stability and association with human diseases, ecDNA molecules may serve as sensitive biomarkers, which will enable early diagnosis. Moreover, as a predictive biomarker, ecDNA might provide patients with instructive guidelines for the choice of chemotherapy, targeted therapy, or immunotherapy, thereby realizing individualized precision medicine. On the other hand, given the important role of ecDNA in cancer progression, developing drugs targeting ecDNA is promising for the future.

Additionally, robust tools are needed. Improving current enrichment and sequencing methods will make the process of extracting circular amplicons more effective. The development of computational or analytical tools such as long-read and single-cell sequencing will facilitate the construction of circular amplicons. As the tools advance, the chromatin structural features of ecDNA will be systematically explored; cancer-associated sequence characteristics of ecDNA will be precisely identified; and a thorough understanding of ecDNA will be gained.

## Abbreviations

EccDNA: extrachromosomal circular DNA
SpcDNA: small polydispersed circular DNA
T-circles/C-circles: telomeric DNA
EcDNA: extrachromosomal DNA
DMs: double minutes
DHFR: dihydrofolate reductase gene
ChIA-PET: chromatin interaction analysis using paired end tag sequencing
BFB: breakage-fusion-bridge
HSRs: homogeneous staining regions
NC: neochromosomes
CBP: histone lysine acetyltransferase CREB binding protein
KDM5B: lysine demethylase 5B
SETDB1: SET domain bifurcated histone lysine methyltransferase 1
FISH: fluorescence in situ hybridization
TEM: transmission electron microscopy
SEM: scanning electron microscopy
AFM: atomic force microscopy
WGS: whole genome sequencing
ATAC-seq: assay for transposase-accessible chromatin with high throughput sequencing
ATAC-see: assay of transposase-accessible chromatin with visualization
GBM: glioblastoma
EGFR: epidermal growth factor receptor
TKIs: tyrosine kinase inhibitors
ALEMs: amplification-linked extrachromosomal mutations
NHEJ: nonhomologous end joining
MARs: matrix attachment regions
HSCC: hypopharyngeal squamous cell carcinoma
HU: hydroxyurea
